# Psychometric validation of the Polish version of the Central Sensitization Inventory in subjects with chronic spinal pain

**DOI:** 10.1186/s12883-021-02510-3

**Published:** 2021-12-11

**Authors:** Barbara Kosińska, Beata Tarnacka, Paweł Turczyn, Grażyna Gromadzka, Małgorzata Malec-Milewska, Dorota Janikowska-Hołowenko, Randy Neblett

**Affiliations:** 1 Department of Neurology and Stroke, District Hospital, Chrzanów, Poland; 2 Department of Rehabilitation, Medical University of Warsaw, Warsaw, Poland; 3 Collegium Medicum, Faculty of Medicine, Cardinal Stefan Wyszynski University, Warsaw, Poland; 4 Department of Anesthesiology and Intensive Care, Medical Center for Postgraduate Education, Warsaw, Poland; 5 Pomorskie Centrum Reumatologiczne im. dr Jadwigi Titz – Kosko, Sopot, Poland; 6 PRIDE Research Foundation, Dallas, TX USA

**Keywords:** Pain, Central sensitization, CSI-pol, Validation

## Abstract

**Background:**

Central sensitization is an amplification of neuronal signaling within the central nervous system. The Central Sensitization Inventory was introduced in 2012. A Polish version of the CSI (CSI-Pol) was developed in 2019, but it was not psychometrically validated. The aim of this study was to validate the CSI-Pol in a sample of Polish-speaking patients with chronic spinal pain and compare them with a group of healthy control subjects.

**Methods:**

The CSI-Pol was administered to 151 patients with chronic spinal pain recruited from two centers. It was re-administered 7 days later. The psychometric properties were then evaluated, including test-retest reliability, construct validity, factor structure and internal consistency. We correlated the CSI-Pol with functional scales, depression and social support scales and compared CSI-Pol scores in the clinical subjects with 30 healthy control subjects recruited from medical staff and their families.

**Results:**

The CSI-Pol demonstrated excellent internal consistency (Cronbach’s α =0,933) and test-retest reliability (Intraclass Correlation Coefficients - ICC =0.96), as well as significant positive associations with other patient-reported scales, including the Neck Disability Index (r = 0.593), Revised Oswestry Low Back Pain Disability Questionnaire (r = 0.422), and other measures of functional and depressive states. An exploratory factor analysis resulted in a 4-factor model. CSI-Pol scores in the clinical sample (35.27 ± 17.25) were significantly higher than the control sample (23.3 ± 8.9).

**Conclusion:**

The results of this study suggest that the CSI-Pol may be a useful clinical tool for assessing central sensitization related symptoms and guiding appropriate treatment in Polish-speaking patients with spinal pain.

**Supplementary Information:**

The online version contains supplementary material available at 10.1186/s12883-021-02510-3.

## Introduction

“Pain is a distressing experience associated with actual or potential tissue damage with sensory, emotional, cognitive, and social components” [1]. Chronic pain has been defined by the International Association for the Study of Pain (IASP) as “pain without apparent biological value that has persisted beyond the normal tissue healing time (usually about 3 months) [2].

Chronic spinal pain is one of the most common problems seen in clinical practice. It is estimated that more than 80% of people will experience low back pain at some point during their lives [3]. Fortunately, most patients recover during the first 3 months or even faster after the first episode of back pain. However, it is estimated that 10–15% of acute pain episodes will develop into chronic low back pain (CLBP) [3]. Neck pain is also a very common musculoskeletal disorder in the general population, with a 1-year prevalence of more than 30% of adults [4].

CLBP is the leading cause of disability and results in more global disability than any other major medical condition in both developed and developing countries [5]. A variety of clinical variables have been found to be associated with CLBP, including: past history of ‘other’ musculoskeletal pain disorders (shoulder, headache, etc.), older age, pain-related catastrophizing, cold hyperalgesia and acute post-traumatic stress responses [6]. Predictive factors for the development of CLBP include: genetic predisposition; psychosocial “yellow flags”, such as catastrophizing, passive pain behavior etc.; increased responsiveness of central and/or peripheral nervous system circuits; and reduced proprioceptive signaling leading to motor and sensory cortical reorganization [7, 8]. Chronic LBP accompanied by insomnia can lead to increased pain intensity [9]. Proinflammatory cytokines and acute phase proteins, including C-reactive protein (CRP) in the central nervous system and circulation, have been implicated in the processes of chronification of pain [10, 11]. In most cases of CLBP, no underlying pathology can be identified [12], which often results in a diagnosis of “non-specific CLBP.”

More recently the role of central sensitization (CS) and other mechanisms have been implicated in chronic spinal pain. Central sensitization has recently been recognized as a pathophysiological mechanism underlying many pain conditions, including fibromyalgia, temporomandibular joint disorder, tension-type headache and chronic spinal pain [13–16]. Various definitions of CS have been proposed as: hyperexcitability of the central nervous system, amplification of neural signaling, hyperexcitement of the central neurons, hyperresponsiveness, and enhanced sensitivity [16]; also many measurement instruments are being used [16)]. Central sensitization is mostly measured with various forms of quantitative sensory testing with conditioned pain modulation tests, functional magnetic resonance imaging, laboratory testing and questionnaires.

The Central Sensitization Inventory (CSI) was developed as a tool to identify patients whose symptoms may be related to CS and/or be associated with a Central Sensitivity Syndrome [17]. The items on the CSI were developed from a careful review of comorbid symptom dimensions among Central Sensitivity Syndromes, which are thought to share a common etiology of CS [17]. These symptoms include widespread pain pattern, sleep disturbance, cognitive slowing, digestive and urological problems, sensitivity to environmental stimuli, etc. Though it does not provide a direct measure of CS, the CSI has been found useful in distinguishing among subject groups with presumably more CS (e.g. fibromyalgia) and less CS (e.g. pain-free control subjects) [17–20]. The inventory has 2 parts. Part A assesses 25 health-related symptoms common to Central Sensitivity Syndromes, with total scores ranging from 0 to 100. Part B (not scored) assesses 10 previously diagnosed Central Sensitivity Syndromes and related disorders. A score of “40” or above, which was initially determined by a receiver operating curve analysis between a subject sample diagnosed with Central Sensitivity Syndromes and a nonpatient comparison sample, has been proposed to indicate the possible presence of CS-related symptomology [21, 22]. It has now been adopted into many languages (available at https://www.pridedallas.com/questionnaires), including Spanish, Italian, Serbian, Japanese, Dutch and others [23–28]. All have demonstrated good psychometric properties [29].

Patients seek treatment in Poland for chronic spinal pain in primary care settings and specialty clinics, such as orthopedists, rheumatologists, neurologists, physiotherapists, and physiatrists. Most of those specialists have not completed extensive specialization in pain management. It is often difficult to classify and differentiate between predominant nociceptive, neuropathic and CS-related pain. A proper identification is essential for optimizing treatment strategies. A Polish version of the CSI (CSI-Pol) was developed in 2019, but it was not psychometrically validated [30]. Therefore the goal of the present study was to validate the psychometric properties of the CSI-Pol (including internal reliability, test-retest reliability, and validity measures) in a sample of patients from a neurological and rehabilitation outpatient clinic who presented with chronic spinal pain in the low back and/or neck and to compare them with a non-patient control sample.

## Methods

In the present study we evaluated the CSI-Pol on a group of chronic spinal subjects and a separate group of control subjects. The study was approved by the Ethical Board of the Warsaw Medical University (Poland) (Consent number: KB/66/2019 obtained 15/04/2019). All participants in this study agreed to participate and signed informed consent forms before the study. All methods were performed in accordance with the relevant guidelines and regulations.

### Participants

We recruited 152 outpatients with chronic spinal pain (some with CLBP only, some with chronic neck pain only, and some with both painful areas, and all with more than 3 months of pain duration) from the Neurological Outpatient Clinic and the Rehabilitation Clinic from National Institute of Geriatrics Rheumatology and Rehabilitation Clinic, Poland. The inclusion criteria were: age between 20 and 80 and chronic spinal pain with more than 3 months of pain duration. We excluded patients with cancer in the brain or in the spine, and other neurological diseases which could cause primary neuropathic pain as polyneuropathies (any causes; patients with diabetes were excluded), no patients had history of heavy alcohol consumption; dementia, previous spine surgery, or recent trauma in anamnesis and poor Polish comprehension skills. Patients with cervical or lumbar herniations with clinical symptoms awaiting surgery, were also excluded. All of the 152 patients completed the CSI-Pol, but one patient was excluded due to incomplete data, leaving 151 patients for analysis, including 24 with chronic neck pain (CNP) and 73 with CLBP and 54 with both CNP and CLBP. The control group consisted of 30 healthy subjects, with no reported spinal pain conditions, who were recruited from medical staff and their families, and agreed complete the CSI-Pol.

### Measures

All of the 151 chronic spinal pain patients completed a battery of patient-reported measures. The 30 healthy subjects completed the CSI-Pol only. Additional demographic data were also collected.

#### Demographic and clinical data

Pain intensity was measured with a Numeric Pain Rating Scale (NRS), from 0 (no pain) to 10 (the worst imaginable pain) over the last 4 weeks. Age and sex of each participant was recorded. Each subject was weighed and their body mass index (BMI) was calculated. Patients were asked in an interview if they were employed, if they used alcohol excessively (over 60 g of 100% alcohol for men and 40 g for women daily) and if they had sleep disturbances (in “yes” or “no” format) caused by pain that prevented them from falling asleep or staying asleep during the night.

#### Central sensitization inventory- polish version (CSI-pol)

The CSI-Pol can be found in Appendix A (Supplement A) and at https://www.pridedallas.com/questionnaires [30]. The CSI consists of two parts: Part A is a 25-item self-report questionnaire which assess health-related symptoms common to CS and Central Sensitivity Syndromes. Each item is rated on a 5-point Likert-type scale (0 = never and 4 = always), with total scores of 0–100 [26]. Part B is not scored. It is designed to determine if the subject has been diagnosed with other CS-related disorders, including restless leg syndrome, chronic fatigue syndrome, fibromyalgia, temporomandibular joint disorder, migraine or tension headaches, irritable bowel syndrome, multiple chemical sensitivities, neck injuries (including whiplash), anxiety or panic attacks, and depression [31].

#### Neck disability index (NDI)

The NDI measures perceived level of disability in subjects with neck pain [32]. The Polish version was used in the present study [33]. The test consists of 10 items concerning various daily activities and other domains and is scored from 0 to 100% disability, with higher scores indicating greater perceived disability [32].

#### Oswestry disability index (ODI)

The Oswestry Disability Index measures perceived level of disability in subjects with low back pain [34]. The Polish version was used in the present study [35]. The test consist of 10 items concerning various daily activities and other domains and is scored from 0 to 100% disability, with higher scores indicating greater perceived disability [32].

#### Clinical psychological diagnostic system. Depression symptoms measurement questionnaire (KPD)

The Depression Symptoms Measurement Questionnaire consists of 75 statements, to which the respondent responds on a 4-point scale. It is used in Poland to assess the symptoms of depression. It contains five problems: Cognitive Deficits and Energy Loss,; Suicidal Tendencies, Pessimism and Alienation; Guilt and Anxiety; Psychosomatic Symptoms and Loss of Interest; Self-regulation. Cognitive Deficits and Loss of Energy.

Scale measures cognitive difficulties such as attention, learning, memory, psychomotor speed, executive functions resulting from depressed mood. Mortality, Pessimism and Alienation Scale examines the subjectively experienced loss of meaning in life, measures the sense of alienation and social isolation. Guilt and Anxiety tension.

concerns feelings of guilt, anxiety, fear, sadness. The items of this scale measure an attitude of dwelling on one’s failures and difficulties. Psychosomatic Symptoms and Decline in Interest measures the subjective evaluation of one’s own health and psychophysical performance. The Self-Regulation scale measures the subject’s emotional and cognitive resources that protect against depression. Depression Symptoms Measurement Questionnaire also has a total score scale measuring overall level of depression which is a sum of scores obtained from individual scales. High scores on these scales indicate high levels of depressed mood symptoms.

#### The Berlin social support scale (BSSS)

The *Berlin Social Support Scale* is a battery of self-report questionnaires developed by Schulz and Schwarzer to measure perceived social support [36]. The Polish version of the BSSS was used in the present study [37].

#### Quantitative sensory testing

We assessed mechanical allodynia using a brush, thermal allodynia using ice in a glove and pinprick hyperalgesia using a wooden cocktail-stick.

### Procedure

All 151 participants completed the CSI-Pol, then completed it again one week later to determine test-retest reliability. Using total CSI-Pol scores, we used the algorithm proposed by Nijs et al. 2015 to differentiate predominant neuropathic, nociceptive and CS pain for each subject [31]. According to their suggestions the neuropathic component of pain was suspected if it was determined to be neuroanatomically logical, with the eventually presence of allodynia, hyperalgesia, with pain characterization of burning, shooting, or pricking (mostly radicular pain). If a neuroanatomically illogical pattern of pain was seen (with or without presence of allodynia and hyperalgesia in these locations), with disproportionate experience of pain to the nature and extent of the injury or pathology (structural impairments which might cause nociceptive LBP) spinal pain with CS was suspected [25]. The diagnosis was made by experienced physicians (neurologists and physiatrists). We divided the patient sample into 5 severity levels groups, as has been recommended previously, to aid the clinical interpretation process, including subclinical = 0–29 points; mild = 30 to 39 points, moderate = 40–49 points; severe = 50–59 points and extreme from 60 to 100 points [22].

### Statistics

Data were analyzed using the statistical package STATISTICA 12.0 (licensed by StatSoft PL, Cracow, Poland) and IBM SPSS Statistics, version 22.0 (IBM, Armonk, NY, USA). Qualitative variables were characterized by the number of important cases (n) and the percentage of the total (%). Categorical comparison of groups was made using two-way tables and chi square test or Fisher exact test. The normality of continuous variables was determined using Kolmogorov–Smirnov. Lilliefors and Shapiro–Wilk tests were used to assess the homogeneity of dispersion from normal distribution.

Variables that were normally distributed were presented as a mean and standard deviation and compared between groups by one-way analyses of variance (more than 3 groups) with post-hoc analysis using Neuman-Keulus test or by student t test (2 groups). A Brown–Forsythe test was used to evaluate the homogeneity of variance (significance < 0.05). Variables that were not normally distributed, or for which the criterion of homogeneity of variance was not completed, were presented as a median and interquartile range (IQR) and compared between groups with Kruskal–Wallis analysis of variance (ANOVAs) (more than 3 groups) with post-hoc testing using Mann–Whitney U-tests with Bonferroni correction of *p* values or by Mann–Whitney U-test (two-way variables). Effect sizes and power analyses were performed using the G*Power 3.1.9.7 tool. Categorical variables were compared between groups using chi2 test or Fisher exact tests. Spearman’s correlation coefficients were used to examine the associations between the Polish version of the Central Sensitization Inventory (CSI-Pol) scores and pain intensity (NRS), NDI and ODI. Correlations were evaluated using the Spearman rank correlation test. The correlation strength was presented according to the Guilford classification [38]:

- Poor when the correlation coefficient r = 0.1 < │r│ ≤ 0.3
- Moderate when 0.3 < │r│ ≤ 0.5
- High (strong) when 0.5 < │r│ ≤ 0.7
- Very high (very strong) when 0.7 < │r│ ≤ 0.9
- Nearly full when 0.9 < │r│ < 1
- Full when │r│ = 1

The criterion for significant differences was *p* < 0.05. Data were given as means (M) with standard deviations (SD). Construct validity and factor structure were determined through the use of questionnaire principal component analysis with Maximum Likelihood Extraction (MLE), with the requirements for extraction being the satisfaction of all three points: scree plot inflection point, Eigen value > 1.0 and accounting for > 10% of variance [39]. The recommended minimum ratio of five participants-per-item was satisfied.

Additionally, the original 4-factor model suggested by Mayer et al. was tested via confirmatory factor analysis (CFA) with ordinal data [17]. In the 4-factor model, Factor 1 originally was named “physical symptoms” and included items 1, 2, 5, 6, 8, 9, 12, 14, 17, 18, and 22; Factor 2 was named “emotional distress” and included items 3, 13, 15, 16, 23, and 24; Factor 3 was named “headache/jaw symptoms” and included items 4, 7, 10, 19, and 20; and Factor 4 was named “urological symptoms” and included items 11, 21, and 25.

Internal consistency of the scale items was determined from Cronbach’s α coefficients [40]. Reliability was determined by test-retest ICC. An error range of 0 ± 10% was allowed in determining the test–retest reliability. The standard error of measurement (SEM) was calculated using the formula: SEM = s√(1 − r), where s = the mean and standard deviation (SD) of Time 1 and Time 2; r = the reliability coefficient for the test and Pearson’s correlation coefficient between test and retest values. Thereafter, the Minimal Detectable Change 90 (MDC90) was calculated using the formula: MDC90 = SEM × √2 × 1.96.

## Results

### Factor analysis

**Fig. 1:**
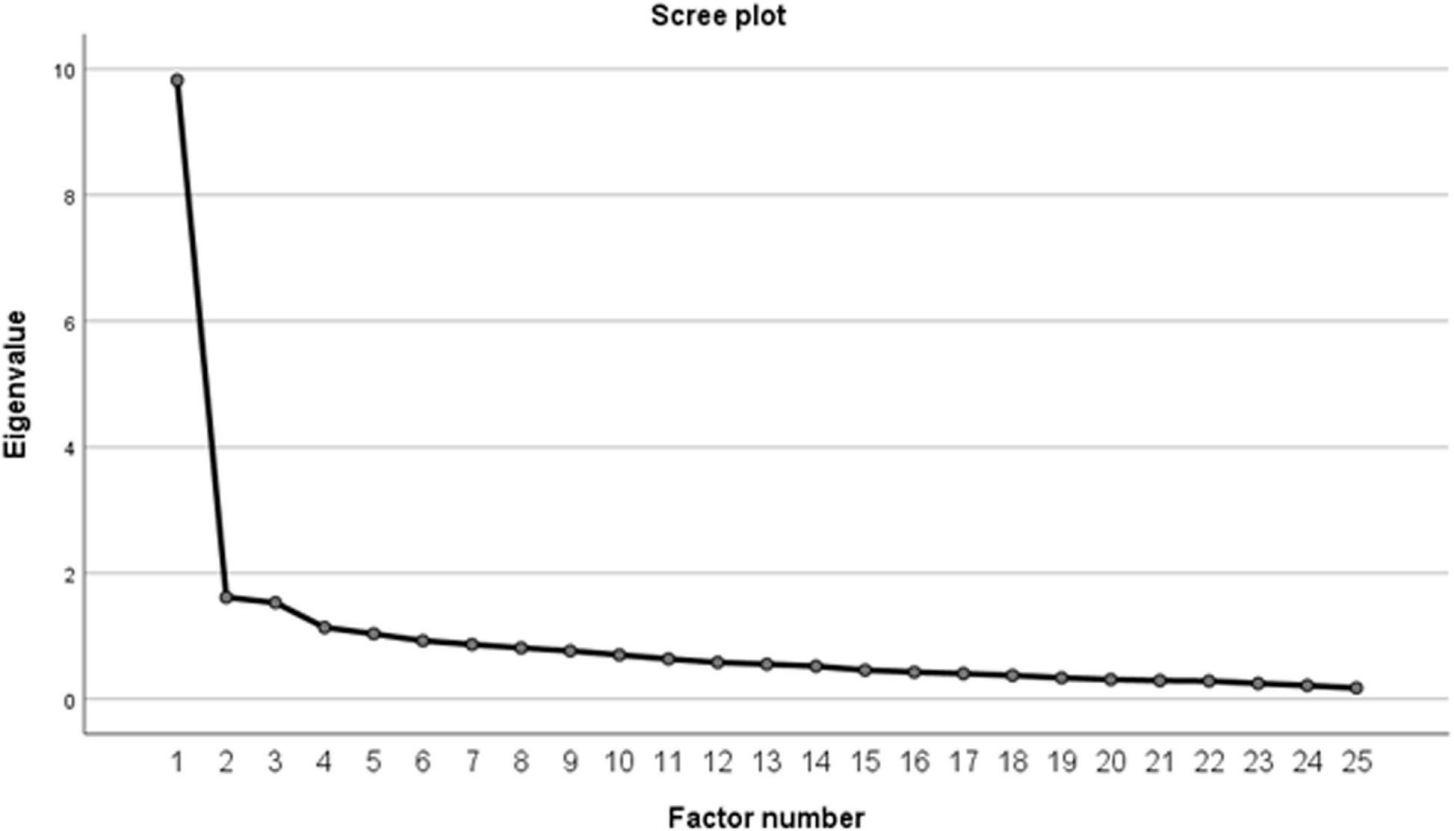
Scree plot from exploratory factor analysis of Polish version of the CSI in patients with chronic spinal pain. Scree pilot indicating one factor solution. (CSI - Central Sensitization Inventory)

The correlation matrix for the CSI-Pol was determined suitable from the Kaiser-Meyer-Oklin values (0.916) and Barlett’s Test of Sphericity (*p* < 0.001). This indicated that the correlation matrix was unlikely to be an identity matrix and, therefore, was suitable for MLE. The factor analysis revealed a satisfactory percentage of total variance explained by the one factor at 39.3%. The CSI-Pol unidimensional was supported by visual inspection of the scree plot, as shown in Fig. 1. The item loading for the one-factor solution for the MLE method and average score for each item is shown in Table 1. The Goodness-of-fit test revealed a Chi square of 529.12 (*p* < 0.000).

**Table 1:** Means (M), Standard Deviations (SD), Corrected Item-Scale Correlations (r_tot_), and Factor Loadings of Both 1-Factor and 4-Factor Models (*N* = 151)

Item	M	SD	r_tot_	1 factor model F^a^	4 factor model			
					F1^b^	F2^c^	F3^d^	F4^e^
1	1.98	1.058	0.619	0.658	0.741			
2	2.22	1.035	0.670	0.686	0.787			
3	0.93	0.974	0.579	0.617		0.666		
4	0.71	1.019	0.513	0.518			0.603	
5	1.19	1.161	0.638	0.638	0.594			
6	0.77	0.921	0.440	0.441	0.406			
7	1.05	1.157	0.529	0.536			0.552	
8	2.25	1.018	0.505	0.505	0.565			
9	1.56	1.173	0.672	0.686	0.725			
10	1.59	1.106	0.518	0.557			0.465	
11	0.74	0.908	0.516	0.495				0.758
12	1.96	1.187	0.625	0.645	0.685			
13	1.25	1.093	0.722	0.763		0.782		
14	1.01	1.126	0.575	0.581	0.509			
15	1.90	1.225	0.680	0.717		0.755		
16	1.57	1.025	0.714	0.770		0.801		
17	1.76	1.109	0.706	0.767	0.709			
18	2.27	1.158	0.564	0.617	0.607			
19	0.66	1.035	0.573	0.595			0.670	
20	0.91	1.107	0.522	0.519			0.716	
21	1.58	1.297	0.507	0.510				0.667
22	1.59	1.216	0.481	0.481	0.509			
23	1.31	1.188	0.677	0.704		0.726		
24	0.67	1.138	0.484	0.497		0.517		
25	1.83	1.294	0.466	0.456				0.349

^a^1-factor model loadings. ^b^Physical symptoms loadings. ^c^Emotional distress loadings. ^d^Headache/jaw symptoms loadings. ^e^Urological symptoms loadings

The rest of the fit indicators suggested that the 1-factor model fit the data satisfactorily (root mean square error of approximation (RMSEA) = 0.08; 90% confidence interval (CI) 0.08 to 0.09). However, the 4-factor model fit the data better than the 1-factor model (RMSEA = 0.07 90% CI 0.07 to 0.08). Standardized factor loadings for the 4-factor model ranged from 0.349 (for item 25) to 0.801 (for item 16). The 4 factors were highly and significantly correlated (r_Factor 1 & Factor 2_ = 0.78; r_Factor 1 & Factor 3_ = 0.70; r_Factor 1 & Factor 4_ = 0.64; r_Factor 2 & Factor 3_ = 0.68; r_Factor 2 & Factor 4_ = 0.56; r_Factor 3 & Factor 4_ = 0.53; *p* < 0.00). Loadings for the single items were identical to those for the 4-factor model (Table 1).

### Reliability - internal consistency and test-retest

Total CSI-Pol scores showed an excellent degree of internal consistency (Cronbach’s α = 0.933) with an individual item range from 0.917 to 0.948. The internal consistency for items in each of the individual factors was somewhat lower. Factor 1 (Cronbach’s α = 0.874), Factor 2 (Cronbach’s α = 0.855) and Factor 3 (Cronbach’s α = 0.734) were acceptable, but Factor 4 (Cronbach’s α = 0.574) was poor.

All patients performed a retest after 7 ± 1 days. The test–retest reliability was high at (ICC = 0.96) with an individual range from 0.74 to 0.91. Measurement error was determined from SEM 0.99 and MDC_90_, being at 0.99 and 2.31%, respectively. Detailed results are in Table 2.

**Table 2:** Intraclass Correlation Coefficients (ICC) and 95% Confidence Intervals (CI) (Lower-Upper Bound) of Test-Retest Reliability

Item	ICC	95% CI
1	0.83	0.773 to 0.874
2	0.837	0.782 to 0.879
3	0.91	0.876 to 0.935
4	0.879	0.837 to 0.911
5	0.88	0.838 to 0.911
6	0.828	0.77 to 0.872
7	0.814	0.752 to 0.861
8	0.785	0.716 to 0.839
9	0.788	0.719 to 0.842
10	0.828	0.77 to 0.872
11	0.753	0.674 to 0.814
12	0.879	0.837 to 0.911
13	0.861	0.813 to 0.897
14	0.804	0.74 to 0.854
15	0.878	0.835 to 0.91
16	0.801	0.735 to 0.851
17	0.742	0.661 to 0.806
18	0.736	0.654 to 0.801
19	0.843	0.79 to 0.884
20	0.886	0.846 to 0.916
21	0.884	0.843 to 0.914
22	0.833	0.775 to 0.877
23	0.874	0.83 to 0.907
24	0.87	0.826 to 0.904
25	0.82	0.759 to 0.866
F1^a^	0.943	0.922 to 0.958
F2^b^	0.95	0.931 to 0.964
F3^c^	0.934	0.911 to 0.952
F4^d^	0.897	0.86 to 0.925
Total CSI-Pol score	0.96	0.945 to 0.971

^a^Physical symptoms loadings. ^b^Emotional distress loadings. ^c^Headache/jaw symptoms loadings. ^d^Urological symptoms loadings. *CSI-Pol* Polish version of the Central Sensitization Inventory

### Comparison of chronic spinal pain subjects with healthy controls

The mean age of the patient population and control sample was 55.7 +/− 14.1 and 42.0 +/− 12.6 respectively. The majority of patients were women (80.1%). The patients differed significantly from controls in age (55.7+/− 14.1 vs. 42.0 +/− 12.6; *p* < 0.001) and sex (women = 80.1% vs. 53.3%) (p < 0.001).

The CSI-Pol scores varied from 0 to 83 points in the total sample, including controls. The total mean CSI-Pol score for all patients and controls combined was 32.8 (SD 16.6). The median was 31.0 (IQR 22.0). The CSI-Pol mean score in the patient sample (35.27 ± 17.25) was significantly different than in the control sample (23.3 ± 8.9). The proportion of patients in each CSI-Pol severity subgroup were: subclinical = 59 (39.1%), mild = 35 (23.2%), moderate = 29 (19.2%), severe = 13 (8.6%) and extreme = 15 (9.9%) [13]. The severity of CSI-Pol total scores was significantly higher in the patient sample compared with the controls (*p* < 0.0003). The most frequent self-reported previously diagnosed CS-related disorder in the patient population, as measured on CSI B, were migraine or tension headaches (*n* = 19; 13%); neck injury, including whiplash (*n* = 20; 13%) and depression (*n* = 22; 15%). In the control sample, migraine (*n* = 3; 10%) and irritable bowel syndrome (n = 1; 3.3%) were the most frequently reported comorbidities on CSI B.

The results of ANOVAs showed that the patients scored significantly higher than the controls on all 4 factors, as shown in Table 3.

**Table 3:** A comparison of 4 Polish version of the Central Sensitization Inventory (CSI-Pol) factor scores in the patient (*n* = 151) and healthy control (*n* = 30) samples

	Patients (mean +/− SD)	Controls (mean +/− SD)	F	Df	P
General disability and physical symptoms	18.2 +/− 7.8 Range: 0–36.0 Median: 19.0 (IQR 12.0)	11.3 +/−4.0 Range 4.0–21.0 Median: 5.0 (IQR 6.0)	^a^22.6	181	< 0.00
Emotional distress	7.5 +/− 5.1 Range: 0.0–21.0 Median: 7.0 (IQR 8.0)	6.1 +/− 3.3 Range 1.0–13.0 Median 5.0 (IQR 6.0)	^a^2.18	181	< 0.14
Headache, jaw symptoms	4.9 +/− 3.8 Range 0–18.0 Median 4.0 (IQR 5.0)	2.8 +/− 2.4 Range 0–9.0 Median 2.0 (IQR 3.0)	^b^χ^2^ 9.5	181	< 0.00
Urological symptoms	4.1 +/− 2.6 Range: 0–10.0 Median 4.0 (IQR 4.0)	1.9 +/− 1.9 Range 0–7.0 Median 1.0 (IQR 3.0)	^b^χ^2^ 12.9	181	< 0.00

^a^The analysis of variance (ANOVA) was applied with post-hoc Tukey analysis. ^b^The Kruskal-Wallis test was applied, and the chi^2^ for the Kruskal-Wallis test is reported here because the assumption of normality of distribution and homogeneity of variances was not met for this factor

### Comparison of chronic spinal pain subgroups (CNP only, CLBP only, and both spinal locations)

The comparison of demographic and clinical variables among three patient subgroups (with CNP, CLBP and both conditions) is provided in Table 4. Compared to patients with only CNP or CLBP, the mean CSI-Pol score was statistically higher in the group with both spinal pain locations (*p* < 0.03). Patients with both spinal locations were also significantly more likely to score above the 40-point CSI-Pol cutoff score (p < 0.03). Mean scores on the NRS pain severity, BMI scale, sleep disturbances, and alcohol use did not differ among the three patient subgroups.

**Table 4:** A comparison of demographic and clinical data among patient subgroups with chronic neck pain (CNP), chronic low back pain (CLBP), and both conditions (*N* = 151)

Parameter	CNP ***n*** = 24	CLBP ***n*** = 73	CNP + CLBP ***n*** = 54	P
**Age, years +/− SD**^**a**^	51.7 +/− 11.9	59.0 +/− 16.7	55.6 +/− 14.6	LS vs. C < 0.07 LS vs. LS + C Ns C vs. L + C Ns
**Women**	22 (91.7%)	55 (75.3%)	44 (81.5%)	Ns
**Men**	2 (8.3%)	18 (24.7%)	10 (18.5%)	Ns
**Pain NRS mean**^**b**^	5.5 (1.5)	6.0 (2.0)	6.0 (2.0)	Ns
**BMI**^**a**^	24.4 +/− 3.6 23.6 (6.4)	27.7 +/− 5.6 26.9 (7.6)	26.1 +/− 4.8 26.0 (5.7)	Ns
**Unemployment**	16 (66.7%)	34 (46.6%)	29 (54.7%)	Ns
**Sleep disturbances**	12 (50.5%)	41 (56.2%)	21 (38.9%)	Ns
**Alcohol use**	0 (0.0%)	10 (13.7%)	6 (11.1%)	Ns
**CSI-Pol < 40**	17 (70.8%)	51 (69.9%)	26 (48.1%)	*p* < 0.03 (whole model) C vs. LS Ns LS vs. C + LS < 0.01 C vs. C + LS < 0.06
**CSI-Pol > 40**	22 (30.1%)	7 (29.2%)	28 (51.8%)	*p* < 0.03 (whole model) LS vs. C ns LS vs. LS + C < 0.001 C vs. L + C < 0.03
**CSI-Pol**	32.0 +/− 16.7 Median 29.5 (IQR 23.5)	31.0 +/− 16.5 Median 32.0 (IQR 25.0)	41.0 +/− 16.5 Median 40.5 (IQR 23.0)	*p* < 0.03 (whole model) LS vs. C ns Ls vs LS + C *p* < 0.01 C vs. LS + C *P* < 0.06
**ODI mean**^**a**^	–	19.7 +/−8.6 Median 19.0 (IQR 12.0) ODI% 39.5 +/− 17.2 Median 38.0 (IQR 24.0)	ODI 21 +/− 7.9 Median 22.0 (IQR 13.0) ODI% 42.1 +/− 15.6 Median 44.0 (IQR 24.0)	Ns
**NDI mean**^**b**^	19.2 +/− 6.2 Median 19.5 (IQR 8.0) NDI% 36.7 +/− 12.9 Median 38.0 (IQR 18.0)	–	19.2 +/− 7.7 Median 20.0 (IQR 11.0) NDI% 38.9 +/− 15.8 Median 41.0 (IQR 22.0)	NS

^a^The analysis of variance (ANOVA) was applied with post-hoc Tukey analysis. ^**b**^The Kruskal-Wallis test was applied with post-hoc Mann-Whitney U test because the assumption of normality of distribution and homogeneity of variances was not met for this factor; categorical variables were compared using chi2 test. *BMI* body mass index, *NDI* Neck Disability Index, *ODI* Revised Oswestry Low Back Pain Disability Questionnaire scale, *CRP* C-reactive protein, *NRS* Numeric Rating Scale, *CSI–Pol* Central Sensitization Inventory, *C* cervical, *LBP* low back pain

### Association between CSI-Pol scores and perceived level of disability

In Table 5, the patients were divided into two subgroups: those who scored below the recommended 40-point CSI-Pol cutoff score and those who scored above [22]. Approximately 40% of patients had a CSI-Pol score above 40, suggesting that their symptom presentation may be related to CS and may indicate the presence of a Central Sensitivity Syndrome. The two groups were then further divided into Neck Disability Index severity subgroups (for the CNP patients) or Oswestry Low Back Pain Disability Questionnaire severity subgroups (for the chronic LBP patients). Compared to those in the below-40 CSI-Pol subgroup, those patients who scored above 40 reported significantly higher levels of perceived disability. On the Neck Disability Index (which offers 4 severity ranges), 82.5% of patients in the above-40 CSI-Pol subgroup, compared with 32% in the below-40 CSI-Pol subgroup, scored in a moderate to severe perceived disability range. On the Oswestry Low Back Questionnaire (which offers 3 severity ranges), 52.6% of patients in the above-40 CSI-Pol subgroup, compared with 29.8% in the below-40 CSI-Pol subgroup, scored in the severe perceived disability range. The correlations between the CSI-Pol and Neck Disability Index in the patient sample was strong (r = 0,593). The correlations between the CSI-Pol and Oswestry Low Back Questionnaire were moderate (r = 0.4222). The most prominent correlations between the CSI-Pol and Neck Disability Index and between the CSI-Pol and ODI were for patients with both CNP and CLBP (r = 0.6663 for NDI and r = 0.598 for Oswestry Low Back Questionnaire).

**Table 5:** a) Neck Disability Index (NDI) and b) Oswestry Low Back Questionnaire (ODI) in patients above and below suggested cut off (*N* = 151)

a)				
Neck Disability Index	% (number) CSI < 40 *n* = 94	% (number) CSI > 40 *n* = 57	p for individual groups comparisons	P for the whole model
Minimal 0–4: no disability	39 (41.5%)	3 (5.3%)	< 0.0000	*P* < 0.00000
mild 5–14: mild disability	25 (26.6%)	7 (12.3%)	< 0.03	
moderate 15–24: moderate disability	26 (27.7%)	27 (47.4%)	< 0.01	
severe disability: > 25	4 (4.3%)	20 (35.1%)	< 0.0000	
b)				
Oswestry Low Back Pain Questionnaire	% (number) CSI < 40 *n* = 94	% (number) CSI > 40 *n* = 57	p for groups comparisons	p for the whole model
Minimal 0–20%: minimal disability	23 (24.5%)	3 (5.3%)	< 0.0024	*P* < 0.00009
Moderate 21–40%: moderate disability	43 (45.7%)	18 (31.6%)	< 0.08	
Severe disability > 41%	28 (29.8%)	30 (52.6%)	< 0.005	

### Association between CSI-pol scores and depressive symptoms

A statistically significant relationship was found between the CSI-Pol and the level of depressive symptoms (Table 6). A positive correlation was found for the general level of depression and all investigated aspects in the Depression Symptoms Measurement Questionnaire (KPD) as Cognitive Deficits and Energy Loss,; Suicidal Tendencies, Pessimism and Alienation; Guilt and Anxiety; Psychosomatic Symptoms and Loss of Interest; Self-regulation. However, the correlation with self-regulation was negative, i.e. in the case of higher values of the Index, lower results of the level of self-regulation were generally observed. The values of the correlation coefficients are presented in Table 6.

**Table 6:** Central Sensitization Inventory CSI-Pol vs Depression Symptoms Measurement Questionnaire – (KPD) and it’s 4 itams measuring symptoms of depressed mood

Parameter	***IOS I (n = 149)***	
	τ	p
Overall result	0,472	< 0,001
Cognitive Deficits and Energy Loss	0,453	< 0,001
Suicidal Thoughts, Pessimism and Alienation	0,437	< 0,001
Guilt and Anxiety	0,385	< 0,001
Psychosomatic Symptoms and Loss of Interest	0,482	< 0,001
Self-regulation	−0,179	0,002

### Association between CSI-pol scores and perceived social support

A statistically significant relationship was found between the CSI-Pol and the perception of overall support measured by Berlin Social Support Scales, as well as the perceived emotional and instrumental support and support seeking with received support and protective buffering. The direction of the correlations were negative. The values of the correlation coefficients are presented in Table 7.

**Table 7:** Central Sensitization Inventory (CSI-Pol) vs Berlin Social Support Scales (BSSS)

	Parameter	***IOS I (n = 151)***	
		τ	p
1.1	Perceived Emotional Support	−0,211	< 0,001
1.2	Perceived Instrumental Support	−0,188	0,002
1.3	Need for support	−0,072	0,218
1.4	Support Seeking	−0,119	0,040
	Actually Received Support, Recipient	−0,121	0,032
	Provided support	−0,117	0,069
	Protective Buffering Scale	0,132	0,022

### Associations between CSI-pol scores and quantitative sensory testing

In 23.17% of patients with a CSI-Pol with chronic spinal pain scores above 40 - widespread, non-neuroanatomical pain was observed, alsoaccording to Nijs et al. guidelines, a disproportion in spinal pain experience was seen [13].

**Fig. 2:**
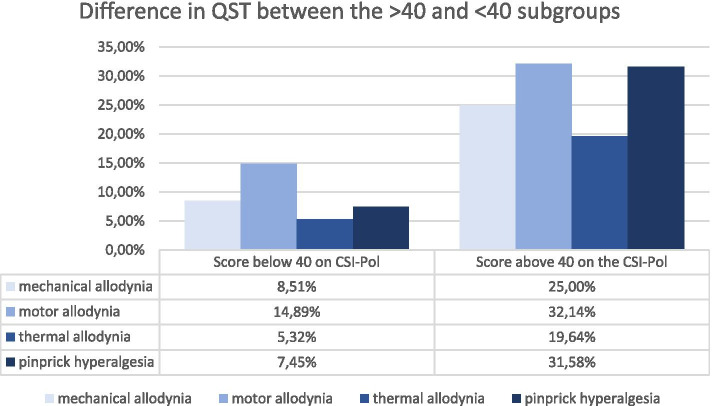
Differences between subgroups in quantitative sensory testing (QST)

The differences in quantitative sensory testing are shown in Fig. 2.

## Discussion

The CSI was developed to screen patients for symptoms related to CS and Central Sensitivity Syndromes so that proper identification and treatment planning can be made. As stated previously, a Polish version of the CSI was published in 2019 (CSI-Pol), but it was not psychometrically validated [30]. Therefore, the aim of this study was to validate the CSI-Pol in a sample of Polish-speaking patients with chronic spinal pain so that it can be made available to Polish physicians, physiotherapist, psychologists, etc. who wish to screen patients for CS-related symptomology.

There are a number of classification algorithms to differentiate nociceptive and neuropathic pain from CS or nociplastic pain, but problems with nosology have been identified [16]. Polish patients with back pain are often transferred from one specialist to another and often undergo physiotherapeutic procedures. They are rarely referred to a psychologist in Poland. The physicians in Poland are not always properly trained in pain management. Identification and categorization of complex pain syndromes, and proper treatment planning, is often difficult, which can lead to unnecessary diagnostic and treatment procedures. Therefore, the CSI is a potential useful instrument to help guide proper assessment and treatment planning for Polish physicians and other health care workers.

Our results showed that the CSI-Pol had excellent internal consistency and test-retest reliability, as well as significant positive associations with functional scales used for CNP and CLBP assessment. The CSI-Pol demonstrated high test–retest reliability in the present study (ICC = 0.96), indicating that it is a reliable instrument. These results are comparable with previous test–retest results of the English [17], Dutch, [23] and Spanish or Serbian, Greek versions [18, 25, 27]. High internal consistency was also found, with Cronbach’s α of 0.933, which is similar to other CSI studies [17, 23, 25, 27]. We confirmed the 4 factor model which was identified by the original developers of the CSI [17]. The internal consistency for Factor 1, Factor 2 and Factor 3 were acceptable, but was poor for Factor 4. Our data are similar to results of the Serbian CSI, where the internal consistency of Factors 1 and 2 was good, and lower internal consistency was found for Factors 3 and 4 [25]. As the authors stated, the lower internal consistency was related to a relatively few number of items in the 3th and 4th factors. In a much larger study, where date were collected from several countries (1987 individuals), the internal consistency was high for (“physical symptoms” 0.88; for “emotional distress” was 0.83), whereas for the other 2 subscales was modest (“headache/jaw symptoms” 0.67 and “urological symptoms” 0.57) [41]. Our data are consistent with the results of the study cited above. The authors stated that that the modest α values obtained in “headache/jaw and “urological symptoms” subscales were expected because Cronbach α is affected by the length of the scale. When subscales are too short the α may be reduced. The multicountry study found that one general “CS-related symptoms” factor was highly reliable, so the authors recommended that only total CSI scores be reported [41].

Significant differences were identified between mean CSI-Pol total scores in the patient population and the control group. The CSI score of 40 out of 100 has previously been shown to be a reasonable cutoff for distinguishing between CSs patients and control subjects. The prevalence of patients in the present study with CSI-Pol scores above 40 was quite high (57 out of 151–38%). Approximately 19% subjects scored in moderate, 9% in severe and 10% in extreme CSI-Pol ranges. We observed significant positive correlations between CSI-Pol scores and self-reported disability, as measured by the ODI and the NDI. Most of our patients who scored below the 40-point cut-off had mild to moderate disability with predominance of minimal disability in ODI and NDI scales. Most patients who scored over 40 reported moderate to severe disability. The correlation between the CSI-Pol and NDI was strong and with the ODI was moderate.

Previous studies with different clinical samples have reported a wide range of scores above 40. For example, in a Japanese study, 11% of musculoskeletal pain patients (not specifically identified as chronic) scored above 40 [22], compared to 58% in a US chronic pain patient cohort who scored above 40 [42]. A range of mean CSI scores have also been identified in different patient populations. For instance, mean CSI scores in an Italian population were 35.2, in Serbian 38.3 and in Dutch were 43.8 [23–25]. A Spanish population (m = 24.6) and a Greek population (m = 29.6) scored somewhat lower [18, 27]. The differenced in mean CSI scores, and the percentage of subjects who have scored above 40, can likely be explained by differences in treatment populations. For instance, the patients in the present study were recruited from an outpatient neurological clinic and a geriatric rehabilitation clinic and not from a specialized pain center, as in the US study.

A large majority of the patients in the present study were women, which is consistent with previous studies on CS-related chronic pain populations [24, 25]. Care-seeking in Poland is more common in women, and in individuals with previous CLBP, poor general health, and with more disabling or more painful episodes [17, 20, 22, 42, 43]. In addition to chronic spinal pain, the most frequent pain symptom reported in our patient population was migraine or tension headaches (reported by 13% and indicated from CSI B). Depression was also reported by 15% of patients on CSI B.

We divided our patient sample into those with CNP and CLBP. In one recent study the median CSI score was 39 in a CNP cohort [44], which was similar to our CNP cohort, which had a median of 35 [44]. This score was below the recommended 40-point cut-off of CSI scale, but is significantly higher than in controls. The mean CSI-Pol score in our CLBP sample was 31.0, which was similar to an Italian LBP population, with a mean of 33.9 [24]. A U.S. patient population with regional lumber pain scored somewhat higher (41.6), which was similar to our group with both pain locations [17]. Similar to our group, the Italian patient group was also recruited from physiotherapy and rheumatology clinics [24]. CSI-Pol values were statistically higher in patients with both pain locations (CNP and CLBP) compared with only one location (CNP or CLBP). The highest score in our patients with both pain locations was expected because higher CSI scores have been observed in previous studies in patients with widespread pain and fibromyalgia [17, 18, 25].

Central sensitization is often associated with mental disorders such as anxiety and depression, and a relationship between chronic pain and depression has validated in several epidemiological studies [45]. Higher CSI-Pol scores in the present study were associated with all the sub-items analyzed in the Depression Symptoms Measurement Questionnaire. Due to the interaction between psychosocial factors and biological mechanisms, it is recommended that CS be considered within the biopsychosocial model. CS-related syndromes share many common features that appear in depressive states, including pain, fatigue, poor sleep, cognitive deficits, headaches, and anxiety, suggesting they may have a common etiology. We recommend therefore to perform the depression assessment in patients with higher CSI-Pol scores.

We found a statistically significant relationship between CSI-Pol scores and perceived social support on several items of the Berlin Social Support Scales. As far as we know, no previous studies have investigated the relationship between the CSI and Berlin Social Support Scales. Higher perceived social support in our patients was related to lower CSI-Pol scores. Social support is very important to reduce suffering. It can reduce the feelings of loneliness in difficult situations, which can significantly improve mood and well-being [46]. The influence of social support is subjects with chronic pain is controversial. Most studies supports our findings that social support in pain patients is associated with less distress, less intense pain, and better overall adjustment [47, 48]. However, there are contradictory findings which show that greater social support can be associated with increased pain severity, perhaps due to reinforcement of pain behaviors by friends and family [49, 50]. When relationships are healthy, we believe that social support can have a more positive than negative influence on pain and on general wellbeing in patients with chronic pain.

The biopsychosocial model assumes that the perception of pain is influences by cognitive, behavioral, emotional, and social components. In this model, it is important to take into account both the biological and social context. For instance, the present study takes into account social support and the aspects of the perception of emotional states affecting the perception and maintenance of pain. These findings in the present study suggest that the experience of symptoms associated with chronic pain and Central Sensitivity Syndromes are multimodal, and require a broader conceptualization than the biomedical model. It is very important that pain physicians not rely on pharmacological interventions alone, but should also utilize biopsychosocial approaches in the treatment o f CS-related symptomology and chronic pain. Available evidence indicates that CS is present in a subgroups especially of the CLBP population and in those patients require intervention targeted at the central nervous system rather than the lower back region. That’s why it’s important to stratify patients into groups with predominantly nociceptive, neuropathic or central sensitization pain in order to schedule the target intervention.

strategies properly. We believe that CSI-Pol will help in this procedure. The main goal of chronic pain treatment should be education to improve the patient’s pain following exercises or daily physical activity. Therefore, pain neuroscience education should be one of the basic therapeutic factors to become not only knowledge for neuroscientists but also for patients to understand their pain. It is important to create a new therapeutic procedures in Poland that would be reimbursed by the state rather than physical therapy procedures.

## Limitations

The current study was performed in one group of chronic spinal pain patients in two neurological and orthopedic outpatient clinics in Poland, so these results may not generalize to other populations.

## Conclusion

This study determined evidence of reliability and validity of the CSI-Pol, suggesting that it may be a useful tool for assessing CS-related symptomology in Polish patients with spinal pain, which may help clinicians in assessment and treatment planning.

## Supplementary Information

**Additional file 1.** CSI-Pol. The polish Central Sensitization Inventory (CSI-Pol) version part A and part B. New corrected version of item 24. Also available at: https://www.pridedallas.com/questionnaires.

**Additional file 2.** CSI-Eng. Original version of Central Sensitization Inventory. Also available at: https://www.pridedallas.com/questionnaires.

## Data Availability

the datasets used and/or analysed during current study are available from the corresponding author on reasonable request.
