# Supplementary material for: Psychometric validation of the Polish version of the Central Sensitization Inventory in subjects with chronic spinal pain
Source: BMC Neurol. 2021 Dec 11;21:483. doi: 10.1186/s12883-021-02510-3 (PMC8665486; doi:10.1186/s12883-021-02510-3)
Supplement: Supplementary file 1 — Additional file 1. CSI-Pol. The polish Central Sensitization Inventory (CSI-Pol) version part A and part B. New corrected version of item 24. Also available at: https://www.pridedallas.com/questionnaires. [file 12883_2021_2510_MOESM1_ESM.pdf]

Indeks Ośrodkowej Sensytyzacji (Część A)

Proszę zaznaczyć odpowiedź najbardziej pasującą do stwierdzenia. Klucz odpowiedzi: Nigdy = 0, Rzadko = 1, Czasami = 2, Często = 3, Zawsze = 4.

1. Czuje się zmęczony i niewypoczęty po przebudzeniu.	Nigdy	Rzadko	Czasami	Często	Zawsze
2. Moje mięśnie są sztywne i obolałe.	Nigdy	Rzadko	Czasami	Często	Zawsze
3. Mam napady lęku.	Nigdy	Rzadko	Czasami	Często	Zawsze
4. Zaciskam zęby lub zgrzytam zębami.	Nigdy	Rzadko	Czasami	Często	Zawsze
5. Mam biegunki i/lub zaparcia.	Nigdy	Rzadko	Czasami	Często	Zawsze
6. Potrzebuję pomocy w wykonywaniu codziennych czynności.	Nigdy	Rzadko	Czasami	Często	Zawsze
7. Jestem wrażliwy na jasne światło.	Nigdy	Rzadko	Czasami	Często	Zawsze
8. Łatwo męczę się podczas aktywności fizycznej.	Nigdy	Rzadko	Czasami	Często	Zawsze
9. Odczuwam ból całego ciała.	Nigdy	Rzadko	Czasami	Często	Zawsze
10. Mam bóle głowy.	Nigdy	Rzadko	Czasami	Często	Zawsze
11. Czuje dyskomfort w pęcherzu moczowym i/lub odczuwam pieczenie podczas oddawania moczu.	Nigdy	Rzadko	Czasami	Często	Zawsze
12. Źle sypiam.	Nigdy	Rzadko	Czasami	Często	Zawsze
13. Mam trudności z koncentracją.	Nigdy	Rzadko	Czasami	Często	Zawsze

14. Mam problemy ze skórą, takie jak: suchość, swędzenie, wysypka.	Nigdy	Rzadko	Czasami	Często	Zawsze
15. Stres pogarsza moje dolegliwości fizyczne.	Nigdy	Rzadko	Czasami	Często	Zawsze
16. Czuje się smutny lub przygnębiony.	Nigdy	Rzadko	Czasami	Często	Zawsze
17. Mam mało energii.	Nigdy	Rzadko	Czasami	Często	Zawsze
18. Czuje napięcie mięśni karku i barków.	Nigdy	Rzadko	Czasami	Często	Zawsze
19. Mam bóle szczęki.	Nigdy	Rzadko	Czasami	Często	Zawsze
20. Niektóre zapachy (np. perfumy) sprawiają że czuję zawroty głowy i/lub mdłości.	Nigdy	Rzadko	Czasami	Często	Zawsze
21. Często oddaję mocz.	Nigdy	Rzadko	Czasami	Często	Zawsze
22. Gdy idę spać odczuwam w nogach dyskomfort i niepokój.	Nigdy	Rzadko	Czasami	Często	Zawsze
23. Mam trudności z zapamiętywaniem.	Nigdy	Rzadko	Czasami	Często	Zawsze
**24. Doznałem/am przeżycia traumatycznego w dzieciństwie**	Nigdy	Rzadko	Czasami	Często	Zawsze
25. Mam bóle w okolicy miednicy.	Nigdy	Rzadko	Czasami	Często	Zawsze
SUMA PUNKTÓW W KOLUMNACH					
	CAŁKOWITA SUMA PUNKTÓW				

Indeks Ośrodkowej Sensytyzacji (Część B)

Czy był/a Pan/Pani diagnozowana z powodu jednej z poniższych chorób?

Zaznacz odpowiedź dla każdej choroby i podaj rok diagnozy.

	TAK	NIE	Rok diagnozy
Zespół niespokojnych nóg			
Zespół chronicznego zmęczenia			
Fibromialgia			
Choroby stawu skroniowo-żuchwowego			
Migrena lub napięciowe bóle głowy			
Zespół jelita drażliwego			
Nadwrażliwość/uczulenie na substancje chemiczne			
Urazy szyi (w tym urazy kręgosłupa szyjnego)			
Zaburzenia lękowe lub napady paniki.			
Depresja			
